# Metacavities by harnessing the linear-crossing metamaterials

**DOI:** 10.1515/nanoph-2024-0443

**Published:** 2025-01-03

**Authors:** Jiaju Wu, Zhiwei Guo, Xin Qi, Qian Wei, Li He, Kai Fang, Yong Sun, Yunhui Li, Yuguang Chen, Haitao Jiang, Hong Chen

**Affiliations:** School of Communication Engineering, Hangzhou Dianzi University, Hangzhou 310018, China; MOE Key Laboratory of Advanced Micro-Structured Materials, 12476 School of Physics Science and Engineering Tongji University, Shanghai 200092, China

**Keywords:** metacavity, linear-crossing metamaterials, negative refraction

## Abstract

The formed optical cavity mode intensively relies on the size and geometry of optical cavity. When the defect or impurity exists inside the cavity, the formed cavity mode will be destroyed. Here, we propose a metacavity consisting of arrays of linear-crossing metamaterials (LCMMs) with abnormal dispersion, where each LCMM offers both the directional propagation channel for all incident angles and the negative refraction across its neighboring LCMMs. Such metacavity can be efficiently excited by a point source, where the excited wave vector components propagate along the same optical path in the cavity. More importantly, the proposed metacavity possesses the remarkable feature of partial defect immunity and geometry robustness. Assisted by two-dimensional transmission lines with loaded-circuit elements, a metacavity with partial defect immunity has been experimentally realized. Our work offers a new avenue for designing optical resonators excited by the point source in integrated photonics, which is very useful for high-efficiency filters, ultrasensitive sensors, and enhancement of light–matter interactions.

## 1 Introduction

Optical resonance cavity is a fundamental optical element of modern photonics, which can be used to efficiently enhance light–matter interaction [1], [2]. As one of the most classically optical resonance cavities, the Fabry–Pérot (FP) cavity has attracted tremendous attention due to its practical applications in a variety of lasers [3], [4], optical switches [5], spectrometers [6], filters [7], and antennas [8]. The physical mechanism of FP-type cavity is the interference of light along a determined optical route, which leads to the formation of cavity mode [9]. Owing to the limitations of the interference conditions for FP resonance, the resonant frequency of cavity mode is strongly dependent on the size and geometry of the optical cavity. Besides, under different incident angles, the light will go different optical routes and the frequencies of cavity modes need shift to meet the requirement of the interference conditions for FP resonance. As a result, the FP cavity would not work under the excitation of an external point source with wave vector components in all directions. Therefore, the plane wave or Gaussian beam with high collimation usually is selected as the excitation source for the FP cavity. This problem also occurs in other types of cavities with photonic walls or barriers in which the cavity mode is formed based on the interference condition, such as photonic crystal cavity [10], [11], [12], [13], whispering gallery cavity [14], [15], waveguide cavity [16], [17], zero-index cavity [18], [19], hyperbolic cavity [20], [21], [22], [23]. Interestingly, some metamaterials supporting negative refraction were proposed to construct another kind of optical cavity which is called open cavity [24], [25], [26], [27], [28]. Based on the mechanism of negative refraction, the formed optical cavity without any walls can be formed. This open cavity can be designed by using two kinds of materials with alternating positive and (effective) negative refractive indices [24], [25], [26]. In 2014, by using three 60° wedges of a photonic crystal with effective negative refractive index, Ge et al. experimentally observed the open cavity mode [27]. Notice that the open cavity mode stems from the formation of a closed path and zero-phase accumulation in the structure, which is different from the conventional cavity modes. As a result, the open cavities can also be excited by a point source, which greatly extends the application scope of the cavity since the point sources are widely used in various systems [29], [30], [31]. Of course, in the open cavity based on the negative-index materials, although the frequency of the cavity mode is fixed, the wave vector components in different directions still go different closed optical paths. As a result, if we put a defect or impurity in the cavity, strong scattering would strongly affect the cavity mode. Up to now, to construct a cavity that can be excited by a point source also has robustness against defects and geometry has remained a challenge.

Recently, hyperbolic metamaterials (HMMs) have improved the ability to precisely tailor the light–matter interactions [32], [33], [34], [35], [36]. By tuning the values of permittivity (*ε*) from negative to positive, the topological transition between dielectric-type and metal-type HMM will occur. At the critical point (i.e., *ε* ∼ 0), a new type of metamaterials possessing two intersecting linear dispersions, called linear-crossing metamaterial (LCMMs) was discovered [31], [37], [38], [39]. Such LCMMs possess an open iso-frequency contour (IFC), which support high-*k* modes similar to HMMs. Furthermore, the permittivity in LCMMs tends to zero, resulting in the zero-phase accumulation along the propagation path just as zero-index materials (ZIMs). Assisted by LCMMs, many interesting applications have been achieved, including angular filters [37], focusing [38], and splitting [39]. Such LCMMs possess unique properties such as negative refraction, which offers a new way to construct LCMMs-induced open cavities. Specifically, the LCMMs also exhibit directional propagating properties under different incident directions, where the incident light from different directions along the same path propagation in the cavity. Therefore, this LCMM may offer a way to realize a metacavity that possesses both geometry independence and defect immunity.

In this paper, we propose a new strategy to construct a metacavity assisted by LCMMs with linear dispersions, where each LCMM offers both a determined propagation channel and negative refraction across its neighboring LCMMs. We show that the LCMMs allow the propagation of light at the same refracted angle even though they are excited under different incident angles. Further, the proposed metacavity possesses a single optical path even if it is excited by a point source. By changing the electromagnetic parameter of the structure, the configuration of the metacavity can also be flexibly designed. More importantly, the designed metacavity has robustness against defects and geometry. Finally, utilizing the arrays of transmission lines (TLs)-based LCMMs, we experimentally demonstrate the realization of such metacavities with partial defect immunity. These findings break the traditional paradigm of optical cavities based on wave-forbidden reflective walls and pave a pathway toward optical resonators excited by the point source.

## 2 Model and method

**Figure 1:**
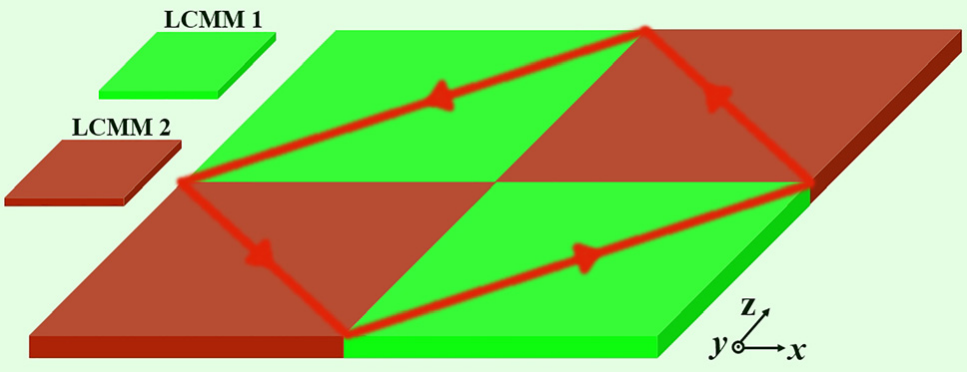
Theoretical model of the proposed metacavity.

Figure 1 shows the theoretical model of the metacavity assisted by arrays of the LCMMs, where a closed optical path can be sustained between the LCMMs under point source excitation. Herein, the red arrows represent the optical route. Different from the usual materials, the LCMM with special dispersion not only supports negative refraction but also exhibits a directional propagating property. Next, we will report a systematic method to construct metacavity assisted by LCMMs. Considering a TE wave propagating in the *xoz* plane, the dispersion relation of a uniaxial material can be described by [32]:

(1) $$\frac{k_{x}^{2}}{\varepsilon \mu_{z}}+\frac{k_{z}^{2}}{\varepsilon \mu_{x}}=k_{0}^{2},$$

where *μ*_*z*_ and *μ*_*x*_ are the permeabilities along the *z* and *x* directions, respectively. Obviously, the iso-frequency contour (IFC) of the material can be flexibly controlled since the electromagnetic parameters (i.e., *ε*, *μ*_*z*_, and *μ*_*x*_) can be separately tuned [40], [41].

**Figure 2:**
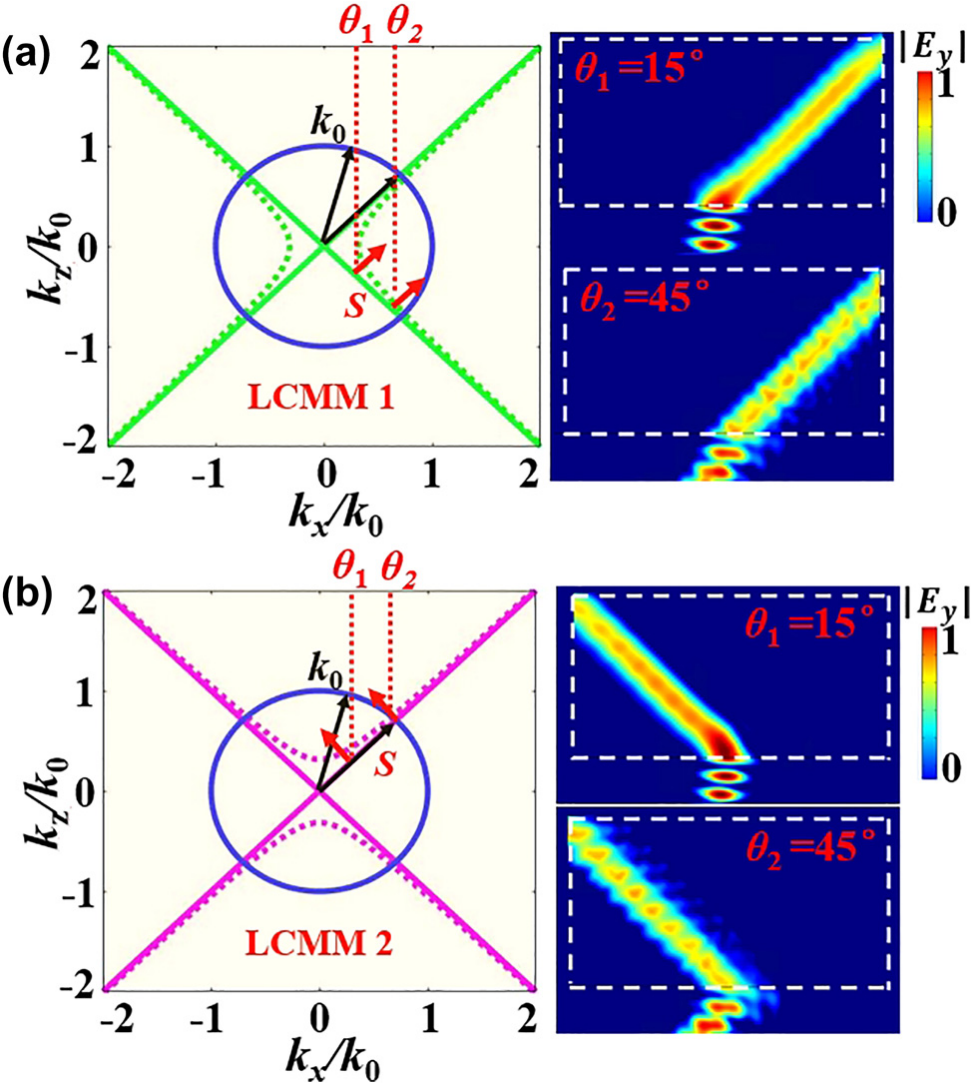
IFC and the corresponding simulated |*E*_*y*_| of the LCMMs with different electromagnetic parameters. (a) *μ*_*z*_ = −1 and *μ*_*x*_ = 1, and (b) *μ*_*z*_ = 1 and *μ*_*x*_ = −1.

To obtain a LCMM, the electromagnetic parameters are first assumed to be *μ*_*z*_ = 1, *μ*_*x*_ = −1 and $\varepsilon =\alpha -\beta^{2}/{\left(2\pi f\right)}^{2}$ , here *α* = 1, *β* = 7.6 GHz, and *f* is the frequency. Notice that such a dispersion of electromagnetic parameters can be realized by 2D TLs with lumped elements [40], [41], [42], [43]. Besides, the permittivity and permeability are relative to the ones of vacuum. When *f* = 1.21 GHz (i.e., *ε* ∼ 0), a linear-crossing IFC occurs which is marked by the green solid lines in Figure 2(a) and the corresponding material is called LCMM1. Here, the blue solid line indicates the IFC of the background material (Air) and the green dashed lines represent the IFC at frequency slightly above the frequency of LCMM1. The black arrows indicate the direction of the incident wave vectors and the red arrows denote the direction of the gradient of wave vectors (i.e., the direction of the energy flow). Based on the boundary condition and causality law, when a wave is obliquely incident into such LCMM, the direction of energy flows is fixed, which are marked by the red arrows in Figure 2(a). The refraction angle is $\theta =\arctan\left(\sqrt{\mu_{z}/\mu_{x}}\right)=45^{◦}$ , which can be tuned by adjusting the relative ratio *μ*_z_/*μ*_*x*_. To intuitively show that the LCMM1 can support a localized mode that has the function of directional transmission, we perform the simulation using COMSOL Multiphysics based on the finite-element method. A Gaussian beam with a finite waist width (*w* = 2*λ*_0_) launches onto the finite LCMM1 from air at different incident angles and the corresponding electric field distributions are given in the insets of Figure 2(a) marked by *θ*_1_ and *θ*_2_. As two cases, we choose 15° and 45° for *θ*_1_ and *θ*_2_, respectively. One can see that the energy flows are positive refractions and the directions of energy flows are fixed under different incident angles. When we only exchange the signs of *μ*_*z*_ and *μ*_*x*_ (i.e., *μ*_*z*_ = −1 and *μ*_*x*_ = 1), the corresponding material becomes another LCMM that is called LCMM2. A linear-crossing IFC still appears that are marked by the pink solid lines in Figure 2(b). The pink dashed lines indicate the IFC at a frequency slightly above the frequency of LCMM2. Interestingly, different from the LCMM1 in Figure 2(a), the direction of energy flow is inverted and the refracted angle is *θ* = −45°. The electric field distributions at different incident angles are given in the inset of Figure 2(b), which clearly shows a negative refraction at different incident angles. It should be pointed out that the direction of the refraction in LCMMs is independent of the incident angles, which is different from the normal refraction realized by conventional metamaterials. The unique properties of LCMMs can be utilized to realize metacavity which possesses the features of impurity immunity and arbitrary configuration.

**Figure 3:**
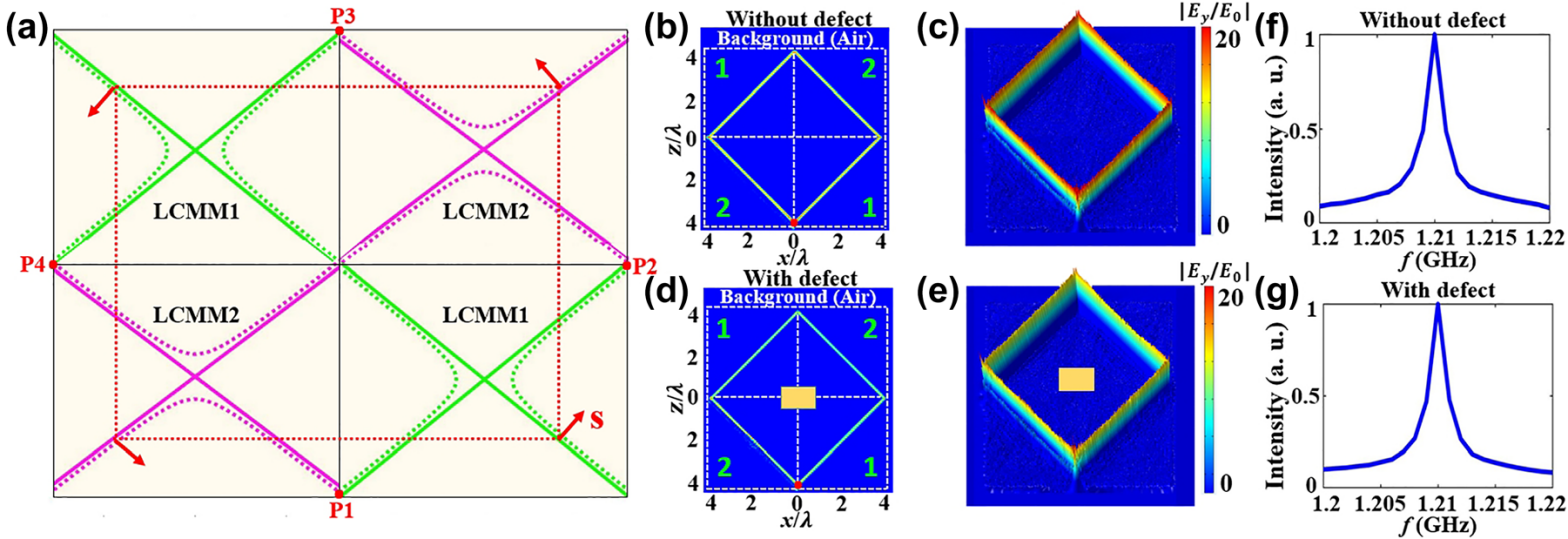
Theoretical design of metacavity. (a) The theoretical design of metacavity via arrays of the LCMMs. The electric field amplitude distributions of the metacavity for different conditions. (b) *μ*_*z*_/*μ*_*x*_ = −1 without defect, (d) *μ*_*z*_/*μ*_*x*_ = −1 with defect. (c), (e) corresponding 3D height of electric field distributions. (f), (g) corresponding the spectral response of the cavity. The yellow regime represents defect.

Figure 3(a) shows the theoretical design of the metacavity via arrays of the LCMMs, where each LCMM offers both the directional transmission channel and the negative refraction of its neighboring LCMMs. The red arrows represent the direction of the energy flow. According to the boundary condition and causality law, when this metacavity is excited by a source (i.e., the point source or Gaussian beam), a closed optical route will be formed. Now, we focus on the case of the point source marked by the red point. In the simulation using COMSOL Multiphysics, the electric field distributions excited by a line current are a standard spherical wave. Thus, the point source can be mimicked by a line current [38], [40], [41], and the value of line current is set to be *I* = 1(*A*). Herein, we set this maximum electric field amplitude generated by the point source in air as *E*0 and normalized it.

Notice that the proposed metacavity can be excited by a point source at some particular positions, while the cavity cannot be excited by point sources at other locations. The detail is given in Supplementary Materials. Considering the equivalence of positions P_1_ to P_4_, we set the point source at P_1_ point in the simulation. The configuration of metacavity and the corresponding full-field simulation are shown in Figure 3(b). Herein, 1 and 2 represent the LCMM1 and LCMM2, respectively. One can see that the cavity can be excited by a point source and the electric field can be enhanced in the structure. To visually see the distribution of the field in the cavity, Figure 3(c) gives the corresponding 3D height of electric field distribution. However, multiple different optical paths are generated when the traditional open cavity is excited by the point source. As a result, the excited electric field will be distributed in the entire structure. If a defect or impurity exists in the cavity, the incident light from a point source will be directly reflected by the defect and the formation of cavity mode will be destroyed. However, the proposed metacavity will not be influenced even when the impurity exists inside the structure as long as the impurity is not placed in the path of propagation channel since the direction of the refracted angle in LCMMs is fixed and independent of the incident angle. In the case of a defect inside cavity, the corresponding 2D electric field amplitude simulation and 3D height of electric field distribution are shown in Figure 3(d) and (e), respectively. Herein, the defects are labeled in yellow. One can observe that such cavity can be excited by a point source operating at a single frequency, and it possesses distinct features including a single light path and partial immunity to impurities. These features arise from the LCMMs support an open and linear dispersion. Interestingly, the ZIMs offer a way to achieve perfect immunity to impurities by using a plane wave under normal incidence, which stems from the zero-phase accumulation within ZIMs [44], [45], [46]. Furthermore, the topological cavities assisted by edge states can support the unidirectional transmission with the capability of defects immunity in a larger bandwidth [47].

To further demonstrate the resonant property of metacavity, Figure 3(f) and (g) give the spectral response of the cavity based on the fast Fourier transform Padé approximation method [48]. It can be seen that the resonance frequency *f*_0_ = 1.21 GHz and the quality (*Q*) factors *Q* = *f*_0_/Δ*f* ≈ 660 are almost unchanged in both two cases with and without defects. Here, the *Q* factors of the cavity modes are finite because there are certain leakages of fields in the propagating path at the interface between the cavity and background. As a consequence, the property of metacavity will not be influenced when the impurity is not placed in the light path in the structure. Besides, such metacavity can be efficiently excited by using either a point source or Gaussian beam. The simulation results under Gaussian beam with different waist widths are shown in Supplementary Materials.

**Figure 4:**
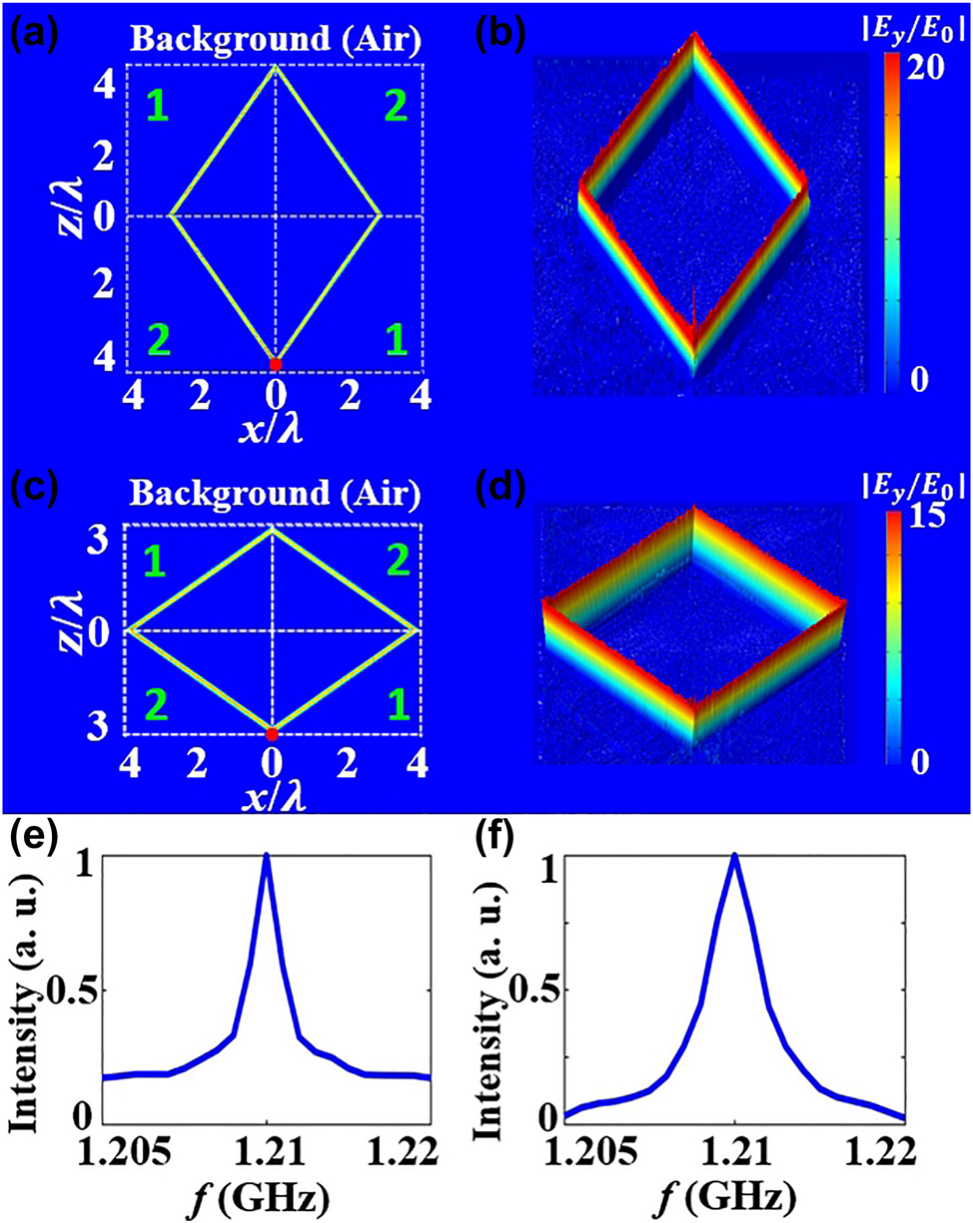
The electric field amplitude distributions of the metacavity for different conditions. (a) *μ*_*z*_/*μ*_*x*_ = −0.5, (c) *μ*_*z*_/*μ*_*x*_ = −2. (b), (d) corresponding 3D height of electric field distributions. (e), (f) corresponding the spectral response of the cavity.

Furthermore, the configuration of metacavity can also be flexibly tuned by changing the relative ratio *μ*_*z*_/*μ*_*x*_ (i.e., adjusting the refracted angle). For example, when *μ*_*z*_/*μ*_*x*_ = −0.5 and keeping the other parameters constant, the corresponding 2D electric field amplitude distribution and 3D height of electric field distribution are shown in Figure 4(a) and (b). It is seen that the shape of metacavity is a parallelogram with the major axis in the *z* direction. The resonance frequency *f*_0_ is 1.21 GHz and the quality factor is *Q* ≈ 850, as shown in Figure 4(e). In case of *μ*_*z*_/*μ*_*x*_ = −2 and keeping the other parameters constant, the configuration of metacavity is a parallelogram with the major axis in the *x* direction and the corresponding full-field simulations are shown in Figure 4(c) and (d). The corresponding spectral response of the cavity is shown in Figure 4(f) and the quality factor is *Q* ≈ 620. It should be noted that the size of the cavity varies owing to the light path generated by the cavity mode in two cases. Thus, the *Q* factors of cavity modes under the two cases are also different [49]. In addition, the influence of the intrinsic loss in materials on the cavity is also given in Supplementary Materials. It should be pointed out that the linear dispersion relationship of the LCMM ensures that the group velocity is perpendicular to the phase velocity, which leads to zero phase accumulation along the propagation path just as in a ZIM [44], [50], [51]. In other words, such metacavity mode is a zero-order FP-type mode. Thus, the formed metacavity mode is independent of structure size at resonance frequency [19].

**Figure 5:**
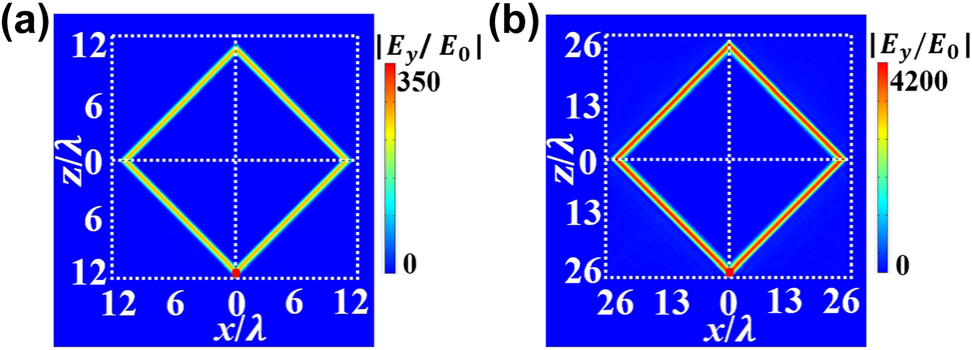
The electric field amplitude distributions of the metacavity for different structure sizes.

More interestingly, the proposed metacavity is similar to whispering gallery-type cavity, and the bigger the structure is, the better field localization would be [49]. From the perspective of defect immunity, the proposed metacavity and other whispering gallery-type cavities both have partial defect robustness when the defect resides away from the light path. Different from whispering gallery-type cavity, the frequency of the formed metacavity mode is independent of structure size. In Figure 5, we show the electric field amplitude distributions of the metacavity for different structure sizes. When the side length of the cavity is 12 times of the incident wavelength, the maximal electric field amplitude is 350*E*_0_, as shown in Figure 5(a). Here, the operating frequency is 1.21 GHz. As the side length of the cavity changes from 12*λ* to 26*λ*, the maximal electric field amplitude rapidly increases from 350*E*_0_ to 4200*E*_0_, as shown in Figure 5(b). Further, we discuss the field distributions excited by point source when the operating frequency is off-resonance, which is given in Supplementary Materials.

## 3 Experimental results and discussion

**Figure 6:**
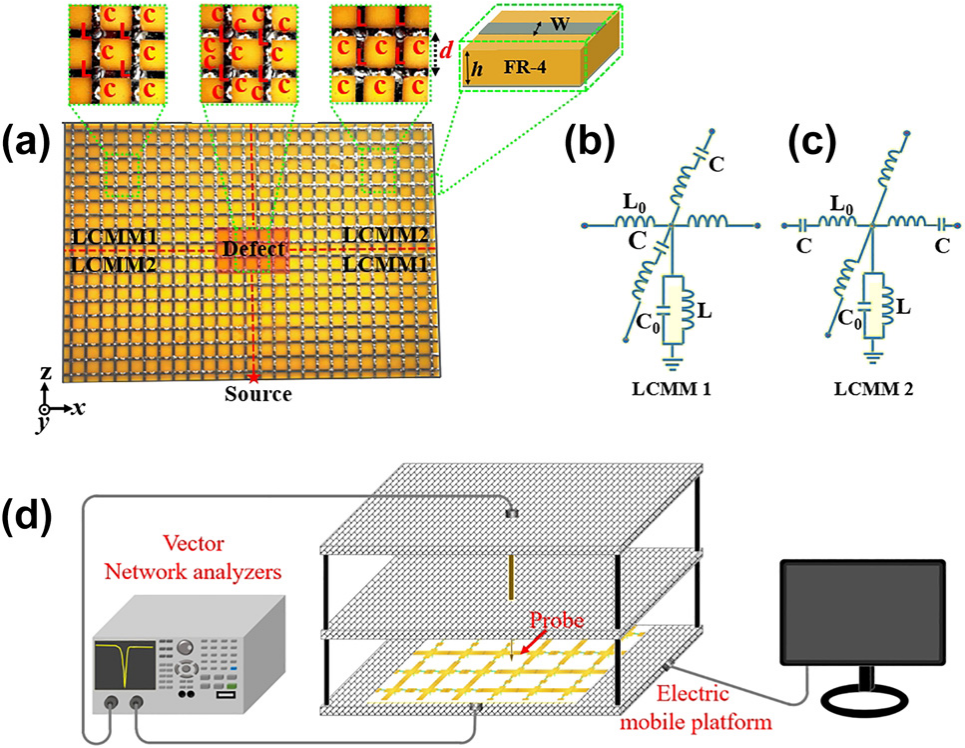
Experimental design and measurement of metacavity. (a) The photo of the TLs-based sample. The defect region is painted in red. The 2D circuit modes for (b) LCMM1 and (c) LCMM2. (d) Schematic view of experimental setup.

Next, we experimentally demonstrate the metacavity can be realized by the effective LCMMs based on the 2D TLs with lumped elements in the microwave regime. The metacavity is composed of arrays of LCMMs supported by a commercial printed-circuit board FR-4 (*ε*_*r*_ = 4.3, tan *δ* = 0.003) with a thickness of *h* = 1.6 mm. The experimental design is similar to the theoretical way and the experimental sample of metacavity is shown in Figure 6(a). The unit cells of LCMMs and defects are shown in the inset of Figure 6(a). The corresponding circuit models of LCMMs are shown in Figure 6(b) and (c). In our experiments, the sample is placed on an automatic translation platform, and the experimental setup is shown in Figure 6(d). In our experiment, the effective LCMMs can be achieved by loading series-lumped capacitors and shunted-limped inductors in to the 2D TLs at the microwave regime. As a matter of fact, because the LCMMs also can be mimicked by a 1D photonic crystal [52] or 2D photonic crystal slab [53], the realization of metacavity can be extended to the optical wavelengths.

To realize LCMMs, the capacitor and inductor are set to 2 pF and 5 nH. For LCMM1 and LCMM2, the capacitors are loaded in the *z* and *x* directions, respectively. The length of unit cell is *d* = 14 mm and the width of the 2D TL is *w* = 2.8 mm. In the circuit model, the structure factor of the 2D TL is *p* = *Z*_0_/*η*_eff_, where *Z*_0_ and *η*_eff_ are the characteristic impedance and effective wave impedance of 2D TL, respectively. If *w* > *h*, the structure factor can be calculated as *p* = 1.393 + *w*/*h* + 0.667 ln(*w*/*h*) + 1.444]^−1^ ≈ 0.255 [40]. The effective permittivity of 2D TLs can be written as:

(2) $$\varepsilon =\left[2C_{0}p-p/{\left(2\pi f\right)}^{2}Ld\right]/\varepsilon_{0},$$

here *C*_0_ represents the per-unit-length capacitance of 2D TL. Based on the quasi-static transverse electric polarized solution and Ampere’s law, the negative *μ*_*z*, *x*_ can be achieved by loading lumped capacitors in the *x* or *z* direction. The relative permeability in the *z* or *x* direction can be defined as:

(3) $$\mu =\frac{1}{p\mu_{0}}\left(L_{0}-\frac{1}{{\left(2\pi f\right)}^{2}Cd}\right),$$

where *L*_0_ represents the per-unit-length inductance of 2D TL. The detailed derivation of the effective parameters is given in Supplementary Materials.

**Figure 7:**
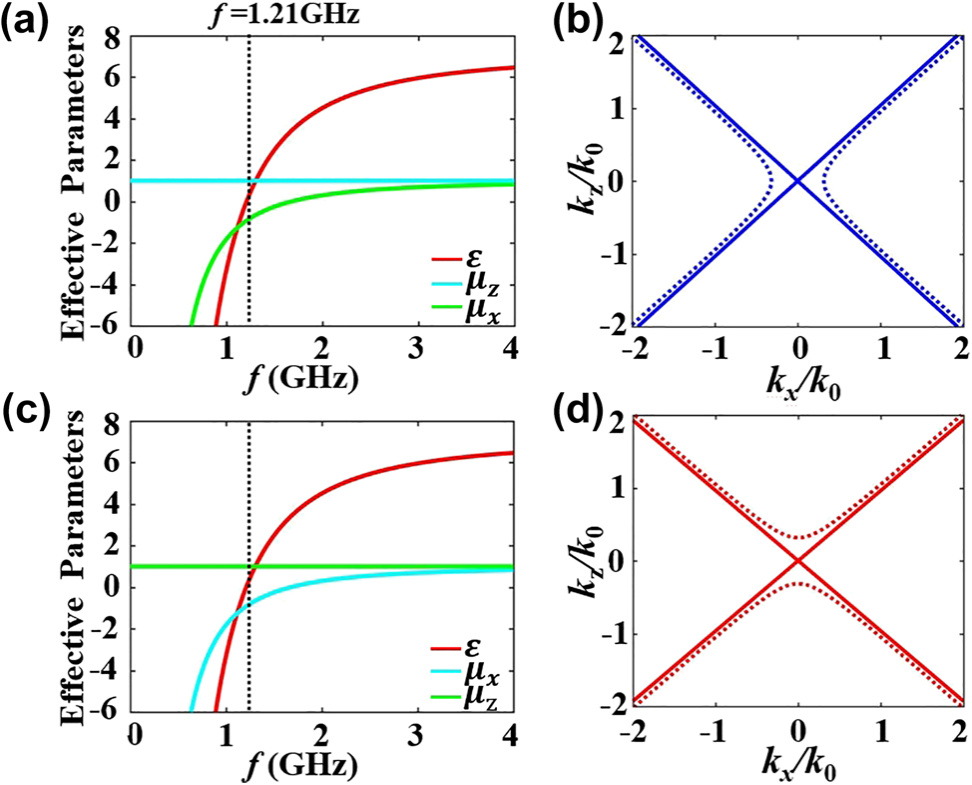
The electromagnetic parameters for (a) LCMM1 and (b) LCMM2. The corresponding IFCs for (c) LCMM1 and (d) LCMM2.

According to Eqs. (2) and (3), the dependence of electromagnetic parameters of LCMMs on the frequency are shown in Figure 7(a) and (c), respectively. The calculated effective electromagnetic parameters for LCMM1 at 1.21 GHz are *ε* ≈ 0, *μ*_*x*_ ≈ −0.93, and *μ*_*z*_ = 1. While the effective electromagnetic parameters for LCMM2 at 1.21 GHz are *ε* ≈ 0, *μ*_*z*_ ≈ −0.93, and *μ*_*x*_ = 1. It should be point out that the permeability in the *z* (*x*) direction is *μ*_*z*_ = 1 (*μ*_*x*_ = 1) since there are no lumped capacitors in the *x* (*z*) direction for LCMM1 (LCMM2). Based on Eq. (1), Figure 7(b) and (d) give the corresponding IFCs of the effective LCMMs. It can see that the characteristic of IFCs for the effective LCMMs are similar to the true LCMMs in Figure 2. Next, we will experimentally report the metacavity can be realized via arrays of the effective LCMMs based on the 2D TLs with lumped elements.

**Figure 8:**
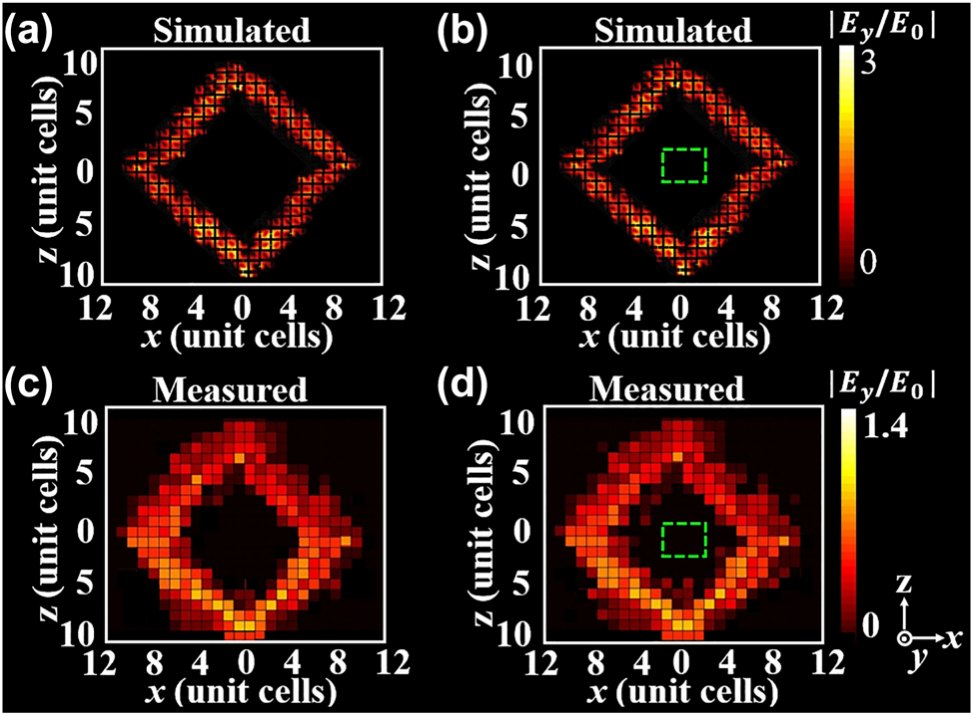
The electric field amplitude distributions at the cross section of the structure (*y* = 0.1 mm). (a) and (c) The simulation and experimental results without defect. (b) and (d) The simulation and experimental results with defect. The green dashed line represents defect.

To prove the metacavity can be realized, the full-wave simulations are performed by CST Microwave studio with open boundary conditions in the *x*, *y* and *z* directions and a discrete edge port as point source is placed into structure [38], [39], [40]. Considering the limited computational ability, we use a finite structure (12*d* × 10*d*) as effective LCMMs, which may introduce some scattering effects due to the constrained structure size. We choose the linear-crossing frequency (1.21 GHz) as the operating frequency to excite the metacavity. A defect is constructed by simultaneously loading the capacitors with *C* = 2 pF in *x* and *z* directions, which can strongly reflect waves. More details can be seen in Supplementary Materials. Figure 8(a) and (b) show the simulated electric field amplitude distributions at the cross section of the structure (*y* = 0.1 mm). It can be seen that the same closed path can be maintained in the cavity even though the electric field distributions are slightly broadened in both two cases with and without defects, as shown in Figure 8(a) and (b), respectively. As a result, a metacavity with both a single path and the defect immunity is realized. Notice that it is necessary to transfer energy through the interface of the structure in order to couple energy into the cavity. Based on the time-domain approach [54], the coupling efficiency is computed to be slightly larger than 65 % in the structure. In the experiment, a vector network analyzer (VNA, Agilent PNA Network Analyzer N5222A) is transported in one 50Ω-subminiature connector, which is placed at the center of the lower edge between LCMM1 and LCMM2. This connector acts as a point source and a small homemade rod antenna of 2 mm is used to measure the out-of-plane electric field |*E*_*y*_| from the cross section of the structure (*y* = 0.1 mm). Figure 8(c) and (d) show the measured electric field amplitude distributions in two cases with and without defects. The defect region is marked by the green dashed line and the number of unit cells in the *x* and *z* directions are also labeled. One can see that the measured results in Figure 8(c) and (d) agree well with the full-field simulations in Figure 8(a) and (b). Besides, the effective parameters of the structure based on the 2D TLs can be actively tuned by loading lumped variable capacitance diodes [41]. Thus, the configuration of metacavity experimentally can also be actively tuned by loading lumped variable capacitance diodes. Up to now, we both theoretically and experimentally demonstrated a method to design a metacavity with partial defect immunity.

## 4 Conclusions

In conclusion, we propose a metacavity consisting of arrays of LCMMs that can be excited by a point source and are not influenced by the size of the cavity. Because of the directional propagating property and the negative refraction property, the excited wave vector components propagate along the same optical path in the cavity. Utilizing the arrays of TL-based LCMMs, we experimentally realize such metacavities with partial defect immunity. Our findings extend the horizons for engineering optical cavities, which can work under point source excitation and has partial robustness against defects and the size of the structure.

## Supplementary Material

Supplementary Material Details
