# Supplementary material for: Metacavities by harnessing the linear-crossing metamaterials
Source: Nanophotonics. 2025 Jan 3;14(1):51–8. doi: 10.1515/nanoph-2024-0443 (PMC11744454; doi:10.1515/nanoph-2024-0443)
Supplement: Supplementary file 1 — Supplementary Material Details [file j_nanoph-2024-0443_suppl_001.docx]

Supporting Information

**Metacavities by harnessing the linear-crossing metamaterials**

*Jiaju Wu, Zhiwei Guo^*^, Xin Qi, Qian Wei, Li He^*^, Kang Fang, Yong Sun, Yunhui Li, Yuguang Chen, Haitao Jiang^*^, and Hong Chen*

Jiaju Wu, Zhiwei Guo, Xin Qi, Qian Wei, Li He, Kang Fang, Yong Sun, Yunhui Li, Yuguang Chen, Haitao Jiang, Hong chen

MOE Key Laboratory of Advanced Micro-Structured Materials, School of Physics Science and Engineering Tongji University, Shanghai 200092, China

Jiaju Wu

School of Communication Engineering, Hangzhou Dianzi University, Hangzhou 310018, China

Zhiwei Guo, Li He, Haitao Jiang

MOE Key Laboratory of Advanced Micro-Structured Materials, School of Physics Science and Engineering Tongji University, Shanghai 200092, China

E-mail: [2014guozhiwei@tongji.edu.cn](mailto:2014guozhiwei@tongji.edu.cn), [hiru@tongji.edu.cn](mailto:hiru@tongji.edu.cn), [jiang-haitao@tongji.edu.cn](mailto:jiang-haitao@tongji.edu.cn)

1. **Discussion of theoretical model and electric field amplitude distributions excited by point sources at different positions**
2. **Metacavity under Gaussian beam excitation**
3. **Influence of loss**
4. **Field distributions of the structure excited by point source at off-resonance frequencies**
5. **Effective electromagnetic parameters for TL model**
6. **Setting of incident field in the simulation within CST**
7. **Discussion of theoretical model and electric field amplitude distributions excited by point sources at different positions**

Based on the boundary condition and causality law, when a point source is placed in the boundary of LCMM, all wave vector components will have directional propagation along two fixed directions and negative refraction across its neighboring LCMMs. Thus, we can predict the optical path when a point source is located in arrays of LCMMs. For example, when a point source is placed at A point [see Fig. S1(a)], all wave vector components will directionally propagate along two fixed directions. Further, the negative refraction occurs at the interface between the LCMMs, while total internal reflection also appears at the interface between LCMM and air. The theoretical predicted optical path marked by the red line is shown in Fig. S1(a). The corresponds to simulated result is shown in Fig. S1(c). Herein, the red point indicates a point source. In this case, the cavity is not excited by a point source since the electric field is not enhanced. Similarly, the cavity cannot be excited by point sources at other locations, such as from point B to H. Next, we further consider another case where the point source is placed inside the structure, as shown in Fig. S1(b). Because the point source is placed at the interface between LCMM 1 and LCMM 2, the wave vector will propagate along two fixed directions in LCMMs, respectively. Here, the yellow and red solid lines represent two different optical paths. The corresponds to simulated result is shown in Fig. S1(d). One can see that the cavity also not be excited by a point source since the electric field is not enhanced. Similarly, the cavity cannot be excited by point sources at other locations, such as from point J to L.


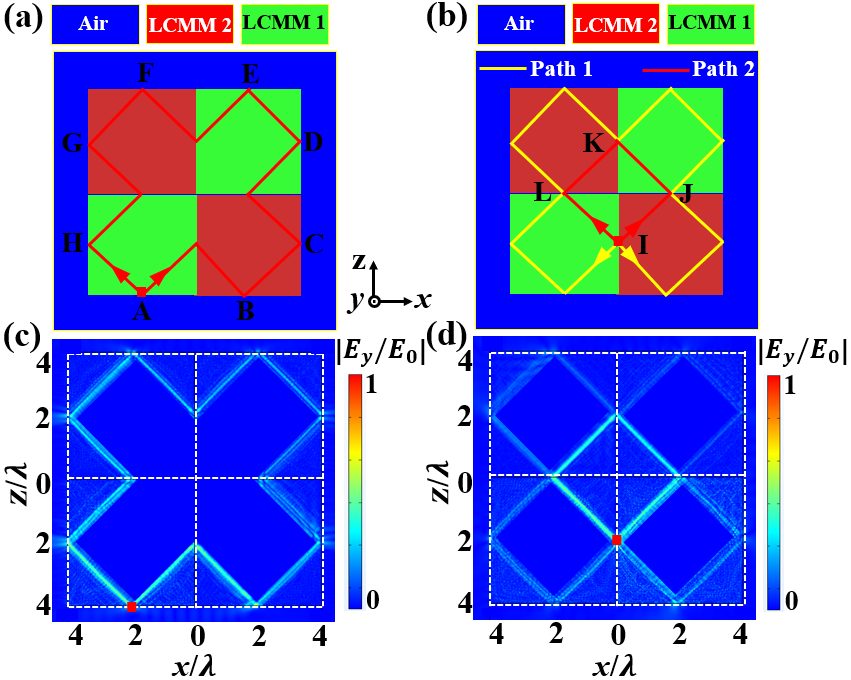


**Figure S1.** Theoretical model of the proposed structure when point source on point A (a) and point I (b). (c) and (d) corresponding electric field amplitude distributions when point source on point A and point I.

Besides, we further consider the situation that the point source is located in different positions along the path of the mode. The corresponds to simulated result is shown in Fig. S2. It can be seen that the closed light path can be formed and the electric fields of the structure are enhanced when the point sources are located in three different positions along the path of the mode. Thus, the metacavity can also be excited when the point source is placed in the path of the mode. These features arise from the LCMMs support an open and linear dispersion.


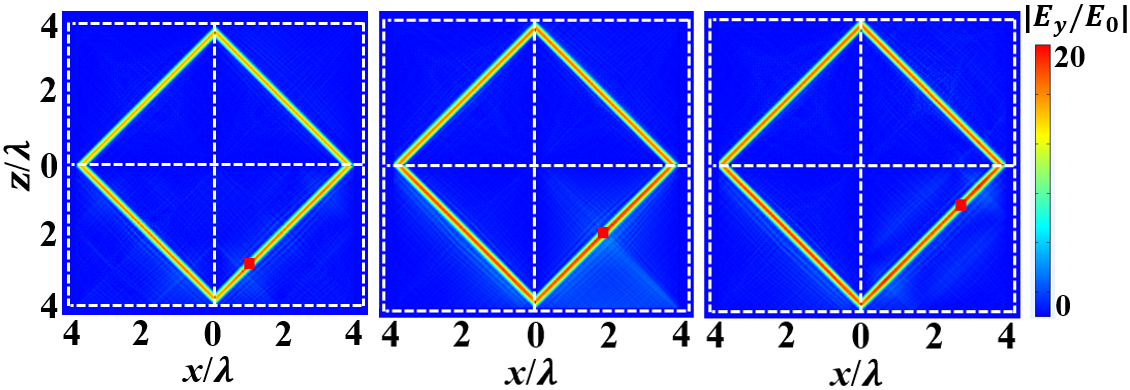


**Figure S2.** The electric field amplitude distributions of the metacavity when the point sources are placed in the path of the mode for three different positions.

1. **Metacavity under Gaussian beam excitation**

To demonstrate that this metacavity can be excited not only by a point source but by Gaussian beam, we give the electric field amplitude distributions of the metacavity under Gaussian beam excitation, as shown in Fig. S3. Here, we consider that a TE-polarized Gaussian beam with different waist width *w* normally launches onto the structure. The electric field of the incident beam can be express as

$E_{y}\left( x,z \right)=E_{0}\exp\left[ -\left( \frac{x}{w} \right)^{2}+i\left( k_{x}x+k_{z}z \right) \right],$ (S2.1)

where *k_x_ = k*_0_sin*θ* and *k_z_ = k*_0_cos*θ* represent the tangential and normal components of the wave vector, respectively. The amplitude of the incident field is set to be |*E*_0_| = 1(*v*/*m*). One can see that such metacavity can be effectively excited by Gaussian beams with different waist widths. As the waist width of the source decreases, the formed optical trajectory becomes narrower. Namely, the size of the optical path can be arbitrarily manipulated by adjusting the waist width of the source. Moreover, we know that the Gaussian beams have angular divergence, such angular divergence will introduce a distribution of the incident angle, leading to the reduction of capability of cavity. The proposed metacavity can overcome this problem because the directions of the excited wave vectors in the cavity are fixed at different incident angles.


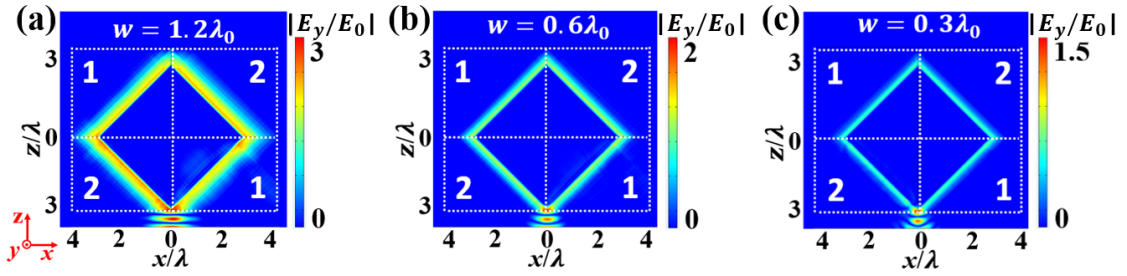


**Figure S3.** The electric field amplitude distributions of the metacavity excited by Gaussian beams with different waist width. (a) *w* = 1.2*λ*_0_, (b) *w* = 0.6*λ*_0_, and (c) *w* =0.3*λ*_0_.

1. **Influence of loss**

Here, we show the influence of the intrinsic loss in LCMM on the cavity. Figure S4 shows the electric field amplitude distributions of the metacavity under point source excitation at different losses. As a common problem, the performance of the cavity decreases as the loss increases. From Fig. S4 (a) to Fig. S4 (c), one can see that the same optical path also appears but the electric field amplitude in the cavity gradually decreases. Here, the red point represents the point source.


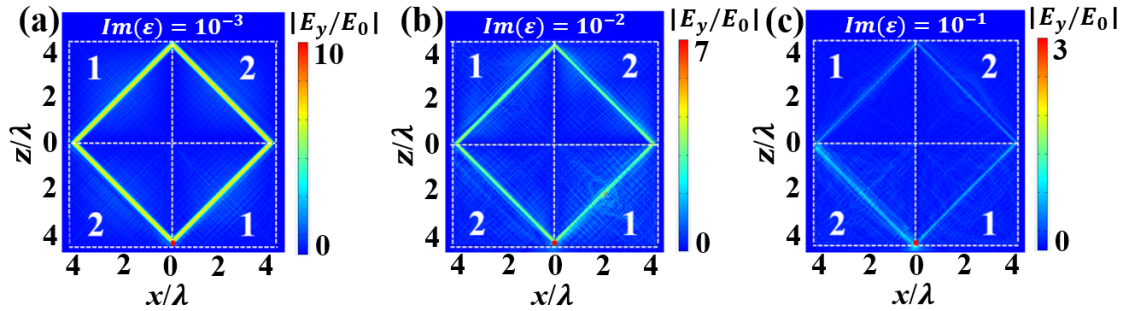


**Figure S4.** The electric field amplitude distributions of the metacavity excited by point source at different losses. (a) Im(ε)=10^-3^, (b) Im(ε)=10^-2^, and (c) Im(ε)=10^-1^.

1. **Field distributions of the structure excited by point source at off-resonance frequencies**

In this part, we discuss the field distributions when the operating frequency at off-resonance frequencies. The effective electromagnetic parameters of structure based on two-dimensional transmission lines with loaded-circuit elements can be calculated by Eqs. (2) and (3). Figures 7(a) and 7(c) show the effective electromagnetic parameters of structure in the manuscript. When the operating frequency is set to 1.23 GHz, the calculated effective electromagnetic parameters for structure 1 are ε ≈ 0.23, *μ_x_* ≈ -0.86, and *μ_z_* = 1. While the effective electromagnetic parameters for structure 2 at 1.23 GHz are ε ≈ 0.23, *μ_z_* ≈ -0.86, and *μ_x_* = 1. The corresponds to the simulated electric field amplitude distribution of the structure excited by point source is shown in Fig. S5(a). In this case, the electromagnetic parameters of structure deviate from the formation conditions of LCMMs. There are multiple light paths and the electric field cannot be enhanced in the structure. When the operating frequency is set to 2 GHz, the calculated effective electromagnetic parameters for structure 1 are ε ≈ 4.5, *μ_x_* ≈ 0.3, and *μ_z_* = 1. While the effective electromagnetic parameters for structure 2 at 1.23 GHz are ε ≈ 4.5, *μ_z_* ≈ 0.3, and *μ_x_* = 1. In this case, the operating frequency is far away from the resonance frequency. The light path is divergent and the electric field is not enhanced in the structure, as shown in Fig. S5 (b).


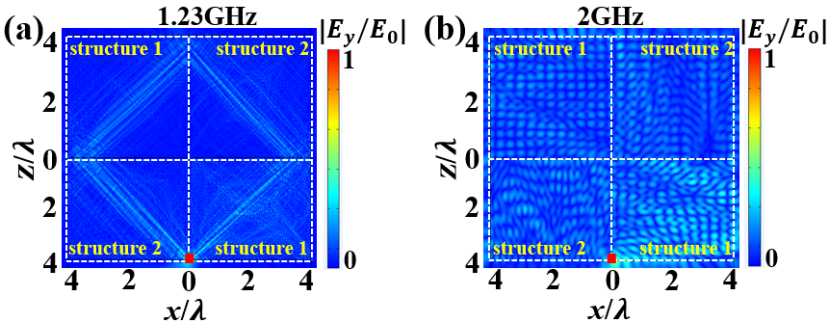


**Figure S5.** The electric field amplitude distributions of the structure excited by point source at different operating frequencies. (a) 1.23 GHz, (b) 2 GHz.

1. **Effective electromagnetic parameters for TL model**

Herein, we give the detailed derivation of the effective electromagnetic parameters. In the circuit-based systems, the relationship between the electric and magnetic fields can be mapped utilizing the relationship between the voltage and the current. Figure S6 shows the TL model of the circuit-based LCMM.


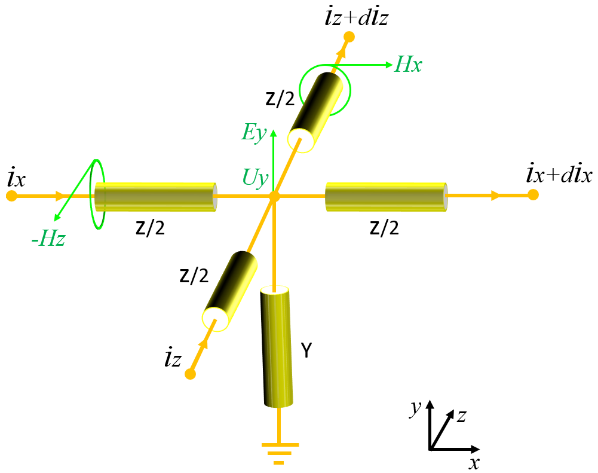


**Figure S6.** TL model of the circuit-based LCMM.

The magnetic fields can be produced by the current from Ampere’s law, as shown in Fig. S6. According to the circuit equation (telegraph equation), we have

$\frac{\partial U_{y}}{\partial x}=-i_{x}Z,$ $\frac{\partial U_{y}}{\partial z}=-i_{z}Z,$ (S5.1)

$\frac{\partial i_{x}}{\partial x}+\frac{\partial i_{z}}{\partial z}=-U_{y}Y,$ (S5.2)

where *U*_y_ denotes the electric potential. Z and Y denote the impedance and admittance of the circuit, respectively. Combining Eq. (S5.1) with Eq. (S5.2), we have

$\frac{\partial^{2}Uy}{\partial x^{2}}+\frac{\partial^{2}Uy}{\partial z^{2}}+\beta^{2}Uy=0,$ $\beta=\pm\sqrt{-ZY.}$ (S5.3)

According to the Maxwell’s equations, the relationship between the electric and magnetic fields can be written as:

$\frac{\partial Ey}{\partial x}=i\omega\mu Hz,$ $\frac{\partial Ey}{\partial z}=-i\omega\mu Hx,$

$\frac{\partial Hz}{\partial x}-\frac{\partial Hx}{\partial z}=i\omega\varepsilon Ey.$ (S5.4)

As a result, by mapping the circuit equation to Maxwell’s equations, the relationship between circuit and electromagnetic parameters can be described by:

$\varepsilon=\frac{Z}{i\omega},$ $\mu=\frac{Y}{i\omega}.$ (S5.5)

Where *ω=*2π*f* is the angular frequency. For the TLs model of the LCMMs in Fig. 6, the impedance and admittance are

$$Z=i\omega L_{0}+1/i\omega C,$$

$Y=i\omega C_{0}+1/i\omega L.$ (S5.6)

Substituting Eq. (S5.5) into Eq. (S5.6), we finally obtain the electromagnetic parameters for the TL model of the circuit-based LCMMs

$\varepsilon=\left( 2C_{0}p-\frac{p}{\left( 2\pi f \right)^{2}Ld} \right)/\varepsilon_{0},$

$\mu=\frac{1}{p\mu_{0}}\left( L_{0}-\frac{1}{\left( 2\pi f \right)^{2}Cd} \right).$ (S5.7)

1. **Setting of incident field in the simulation within CST**

In CST Microwave studio, the point source can be mimicked by a discrete edge port. To demonstrate this point, we simulated the electric field distribution excited by a discrete edge port inside the structure. Herein, the parameters and design of model agree with Fig. 6 in the manuscript. Figure S7 gives the electric field amplitude distributions, where loading the capacitor and inductor are equal to zero. In this case, the structure can be seen as a uniform material. One can see that the electric field distributions excited by a discrete edge port are a standard spherical wave, as shown in Fig. S7(a). Thus, this discrete edge port can be seen as a point source in the simulation [1-3]. Besides, we set this maximum electric field amplitude generated by this point source in the structure as E0 and normalized it. Next, we further reveal the proposed defect will cause strong scattering. The design of defect is shown in the inset of Fig. 6(a) in the manuscript. It is seen that the simulated electric field amplitude distributions change drastically when this defect lies inside the structure, as shown in Fig. S7(b).


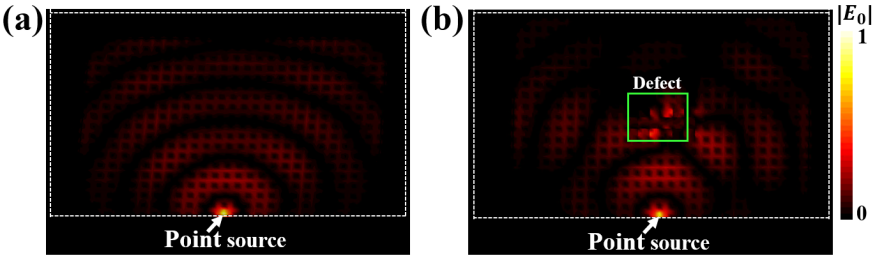


**Figure S7.** The simulated electric field amplitude distributions. (a) without defect, (b) with defect.

**References**

[1] K. Yu, Z. W. Guo, H. T. Jiang, and H. Chen, “Loss-induced topological transition of dispersion in metamaterials,” J. Appl. Phys., Vol. 119, p. 203102, p. 2016.

[2] Z. Guo, H. Jiang, K. Zhu, Y. Sun, Y. Li, and H. Chen, “Focusing and super-resolution with partial cloaking based on linear-crossing metamaterials,” Phys. Rev. Appl., Vol. 10, p. 064048, 2018.

[3] Y. Long, J. Ren, Z. Guo, H. Jiang, Y. Wang, Y. Sun, and Hong Chen, “Designing All-Electric Subwavelength Metasources for Near-Field Photonic Routings,” Phys. Rev. Lett., Vol. 125, p. 157401, 2020.
